# A survey of biclustering and clustering methods in clustering different types of single-cell RNA sequencing data

**DOI:** 10.1093/bfgp/elaf010

**Published:** 2025-08-10

**Authors:** Chaowang Lan, Xiaoqi Tang, Caihua Liu

**Affiliations:** School of Artificial Intelligence, Guilin University of Electronic Technology, Guilin 541004, Guangxi, China; School of Artificial Intelligence, Guilin University of Electronic Technology, Guilin 541004, Guangxi, China; School of Artificial Intelligence, Guilin University of Electronic Technology, Guilin 541004, Guangxi, China

**Keywords:** scRNA-seq, clustering, biclustering, dataset properties

## Abstract

Single-cell RNA sequencing (scRNA-seq) technology has garnered considerable attention as it enables the exploration of cellular heterogeneity from a single-cell perspective. Various unsupervised methods, such as biclustering and clustering methods, offer a theoretical foundation for understanding the structure and function of cells. However, accurately identifying cell subtypes within complex scRNA-seq data remains challenging. To evaluate the current development status; summarize the strengths, weaknesses, and improvement strategies of unsupervised methods; and provide guidelines for future research, we surveyed five biclustering and 21 clustering methods applied to different types of scRNA-seq datasets. We employed three external and two internal metrics to determine clustering performance on 10 publicly available real datasets. Dataset properties are quantified from six perspectives to discover the most suitable biclustering or clustering methods. The results of this survey indicate that biclustering methods are effective for identifying local consistency or for deeply mining partially annotated datasets. Conversely, clustering methods are more suitable for dealing with unknown datasets. This survey aids in identifying cellular heterogeneity by recommending appropriate methods based on different dataset characteristics.

## Introduction

In the past decade, RNA sequencing (RNA-seq) technology has become an essential tool for cell population studies [1]. However, RNA-seq, which measures average gene expression values from mixed RNA samples, neglects intercellular heterogeneity [2]. Take tumor as an example: due to complex biological processes and environmental perturbations, primary tumor cancer cells, metastatic lesions, and *in situ* and peri-cancerous areas exhibit significant differences in gene expression [3]. RNA-seq technology measures the average gene expression in groups of cancer cells, but does not analyze gene expression levels of individual cells [4]. Single-cell RNA sequencing (scRNA-seq) technology has been developed to overcome this limitation and has provided a new direction to analyze the behavior, mechanisms, and relationships of individual cells [5].

ScRNA-seq enables the analysis of individual cells by transcribing RNA into complementary DNA (cDNA), then using second-generation sequencing of this cDNA to capture expression information [6–12]. In general, scRNA-seq has gained popularity in bioinformatics due to specific kinds of contributions it can make to biological and medical research. In biology, scRNA-seq can help researchers describe cellular heterogeneity in many ways, including identifying new cell types (<1%), adding markers to known cell types, predicting cell traces, and elucidating regulatory networks. It is also especially useful in oncology research. Notable examples include improving the molecular understanding of malignant cells in lymphoma [13], researching intra- and inter-tumor heterogeneity in cancer populations under drug treatment [14], and performing fuzzy diagnosis modeling tumor markers as a starting point [15]. In medical research, scRNA-seq benefits clinical diagnoses by permitted detailed analysis capable of identifying complex disease mechanisms. For instance, one recent scRNA-seq study discovered that the true source of metastatic recurrence of colorectal cancer was a population of residual EMP1-positive cells. This finding provided new clinical treatment options for rectal cancer patients [16].

Although scRNA-seq offers new technological support for cytological and biomedical exploration, it also has many shortcomings. For example, due to the extremely small number of transcripts within a single cell, the low efficiency of messenger RNA capture [17] and unregulated objective reasons in the experimental environment can result in high-dimensional, sparse, excess noisy, and large-scale sequencing data. These poor-quality data have a significant negative impact on subsequent downstream analyses.

Clustering is an initial phase of scRNA-seq analysis and is very critical aspect in subsequent downstream analyses. It aims to identify different cell types through maximizing the similarity among cells within the same cluster while minimizing dissimilarity among different clusters [18]. Clustering can also be used to reconstruct cell developmental trajectories [19], discover rare cells [20], and build spatial models of complex tissues, which in turn can contribute to an improved understanding of the basic biology and disease etiology [21].

The biclustering method, adeptly capturing intricate mechanisms at both cellular and genetic levels, is also a subsequent downstream analyses of scRNA-seq data. This method considers the similarity between genes and cells, emphasizing their concurrent clustering, potentially yielding high precision. However, this method is also complex and time-consuming.

While both clustering and clustering methods exhibit good performance, few aggregated and systematic comparisons between them have yet been performed, thus hindering an effective understanding of their relative strengths and limitations. Our study addresses this gap via a systematic review of state-of-the-art biclustering (five methods) and clustering (21 methods). We provide a comprehensive overview of each method’s core workflow and preprocessing steps. Three external metrics and two internal metrics were employed to evaluate performance, with runtime analysis included. A total of 10 scRNA-seq datasets spanning diverse tissues and dimensional scales were rigorously selected, thereby enabling a multimetric assessment in six evaluation dimensions to quantify dataset-method compatibility. This approach allows for an exploration of how variations in dataset can impact clustering results, an aspect often overlooked by other reviews. In developing this comparison, our aims are as follows:

- *To identify the most suitable method for different types of datasets and help users choose the correct method for their datasets.*
- *To summarize the strengths and weaknesses of biclustering and clustering methods, and provide suggestions for future improvement.*
- *To offer insights and recommendations for future clustering research.*

## Methods

Biclustering and clustering are two popular methods for identifying cell groups in scRNA-seq data. In this chapter, we give a brief introduction to both methods.

### State-of-the-art biclustering methods for clustering ScRNA-seq data

Here we introduce five types of biclustering methods: graph-based biclustering methods, information-theoretic biclustering methods, long common subsequence alignment-based biclustering methods, statistical-based biclustering methods, and factor decomposition-based biclustering methods.

#### Graph-based biclustering

The graph-based biclustering method is grouping cells via graph-clustering method. BiSNN-Walk [22] is a famous graph-based biclustering method that iteratively performs cell clustering and filters out candidate genes. Cell clustering is performed by constructing a shared nearest neighbor (SNN) graph, followed by applying the Walktrap method [23] to cluster the SNN graph. The strategy for selecting candidate genes involves retaining only those genes whose median expression level within a specific cell cluster is higher than the 75th percentile of that gene’s overall expression level across all other cell clusters.

#### Information-theoretic biclustering

QUBIC2 [24] is designed to detect functional gene modules, also known as biclusters, via a three-step process rooted in information theory. First, a left-truncated Gaussian mixture model is employed to discretize the gene expression matrix. This transforms it into a binary matrix [2]. Second, the gene pairs in the binary matrix are sorted in descending order based on their weight. Next, genes that are highly correlated with gene pairs are selected by their Kullback–Leibler divergence score—an information-theoretic metric—to form initial core clusters. Third, we extend these core clusters horizontally and vertically to find the intersection between these expansions, termed ”dual clusters.” The output is derived from both the core and dual clusters.

#### Long common subsequence alignment-based biclustering

runibic [25] is a biclustering method designed with the R package. This method has four steps. The first step involves generating an index matrix. The second involves using the Longest Common Subsequence (LCS) method to identify seeds from the index matrix, then sorting these seeds. Next, ordered bimodules are identified by extending these seeds and repeatedly applying the LCS method. Finally, the LCS method is used to determine the scope of the expanded clusters.

#### Statistical-based biclustering

GiniClust3 [26] uses two statistical measurements—i.e. the Gini index and the Fano factor index—to group cells. These processes comprise three components. The first is the Gini index-based clustering component, which calculates the Gini index for each gene to construct a high Gini-gene expression matrix. Next, it arctan-transforms the matrix to generate a transformed expression matrix to which the Leiden method is applied to generate Gini clusters. The second component is a Fano factor-based clustering component, which calculates the Fano factor, then selects genes with high Fano factors to form a high Fano-gene matrix. The Leiden method is then used again to produce Fano clusters. Interestingly, both Gini index-based and Fano factor-based clustering components can operate simultaneously. A final component is then used to simplify the consensus matrix generated by the Gini and Fano clusters to generate a simplified consensus matrix. Finally, a k-means method is used to derive final clusters.

#### Factor decomposition-based biclustering

The foundational mechanism of the SSLB method [27] is factor decomposition. This process involves extracting desired clusters from gene expression matrices via factor decomposition, which can be dynamically adjusted using a scale factor.

### State-of-the-art clustering methods for clustering scRNA-seq data

More recently, graph clustering and deep-learning-based approaches have emerged as the state-of-the-art protocols for performing analysis on scRNA-seq datasets. Graph clustering is an extension of density-based clustering, in which each cell is treated as a data point, and the similarity in gene expression among cells is considered as an edge weight between data points. Graph clustering can easily represent complex nonlinear structures, but its performance can be heavily impacted by the reliability of the similarity matrix [28]. Since the resounding success of deep learning in the 2012 ImageNet competition [29], researchers in various fields have applied deep learning methods to a wide variety of fields due to innate advantages. Bioinformaticians have recognized the potential of deep neural networks and have used them to perform data representations and to conduct clustering analyses of structures, thereby enabling the handling of more complex and greater-dimensional data [30]. In addition, many studies have explored combining graph clustering with deep learning-based clustering approaches, while both consensus and hierarchical clustering are also widely used.

#### Graph clustering

Seurat is a popular graph clustering tool that first appeared in 2015 [31]. Here, we used version 4.3.0 to conduct comparative experiments [32]. This method initially constructs a weighted nearest neighbor (WNN) graph to measure the extent of neighborhood overlaps between every pair of cells. Subsequently, this method uses these extents as edge weights to construct an SNN graph. Next, it employs a smart local moving method at a high resolution (resolution = 5) to merge clusters that lack clear evidence of separation.

ScGSLC [33] is a graph-based similarity learning method that integrates scRNA-seq data with protein–protein interaction networks into a single graph. Here we used a graph convolutional network (GCN) to embed the graph and to cluster cells based on the computed intergraph similarity.

MPSSC [34] is a spectral clustering method based on learning similarity matrices. In general, spectral clustering is a special form of graph-based clustering. Unlike traditional graph clustering methods, MPSSC uses the Laplacian matrix of the graph to perform clustering. MPSSC improves upon ordinary spectral clustering methods by combining multiple similarity matrices, introducing sparse structure constraints, and shrinking inter-row differences. These modifications optimize its performance when handling high noise and missing data.

#### Deep learning-based clustering

ScSSA [35] consists of two dimensionality reduction steps and a clustering step. First, a semisupervised deep counting autoencoder is used to perform the initial dimensionality reduction. Second, a fast independent component analysis (FastICA) method was used for subsequent dimensionality reduction. Finally, a Gaussian mixture model was used for the cluster cell.

ADClust [36] is another Gaussian-based deep-embedded clustering method that involves two modules: i.e. an autoencoder module and clustering optimization. The autoencoder module is designed to learn deep embedding representations of cells. Next, the clustering optimization module employs a multipart strategy that combines the Louvain method [37] with the Dip-test method [38] to cluster cells based on the learned embedded cell representations.

DeepScena [39] is a deep learning clustering method based on the ZINB distribution, and consists of two main modules. The first is an NB-based convolutional autoencoder that is used for data denoising, dimensionality reduction, and initial clustering. The second module, MNet, is a fully connected pairwise similarity-enhancing network that refines clustering procedures by using pairwise cell similarities within a reliable subspace to self-supervise training processes.

scDECL [40] is a deep enhanced constraint clustering algorithm that leverages contrastive learning. First, it applies contrastive learning, pretext task learning, and a mixup data augmentation strategy to develop and learn a latent feature space. Next, it constructs pairwise constraints by incorporating prior label and pairwise distance information from cells. These are then combined into enhanced pairwise constraints to refine the clustering process.

SCLEGA [41] uses a ZINB-based model for dimensionality reduction and denoising. It employs a Graph Autoencoder method based on GCNs to construct a graph and get a latent space. Initialization cluster centers are determined by the Leiden algorithm, and cells are adaptively labeled based on their positions in the latent space.

#### Combining graph clustering with deep learning-based clustering

scGNN2.0 [42] is a graph neural network-based method that incorporates a hybrid iterative circle that consists of three stacked autoencoders: a feature autoencoder, a graph attention autoencoder, and a cluster autoencoder. The feature autoencoder is responsible for feature selection, while the graph attention autoencoder handles dimensionality reduction. The cluster autoencoder first uses Louvain [43] clustering to estimate k, the number of clusters, then applies traditional k-means clustering. The output of the cluster autoencoder serves as an input for the feature autoencoder, thereby creating a closed-loop architecture.

AttentionAE-sc [44] combines a ZINB-based autoencoder method for denoising with a graph autoencoder-based method for dimensionality reduction using an attention mechanism. Iterative fusion outputs are then self-optimized for clustering via the Leiden [45] and DEC [30] protocols, respectively.

#### Consensus clustering

SC3 [46] is a consensus clustering method that combines the results of multiple clustering algorithms, including k-means and hierarchical clustering, to generate a consensus matrix. It then extracts final clustering results via spectral clustering.

SCENA [47] constructs multiple cell-to-cell similarity matrices by selecting gene sets that characterize the relationships between cells. Clustering is then performed on each similarity matrix, and the resulting clusters are combined into a single consensus matrix. Next, a local affinity matrix is employed to strengthen the similarity of results within cell groups, and clustering results are then iteratively refined.

#### Hierarchical clustering

scHFC [48] combines the Simulated Annealing and Genetic Algorithm optimized Fuzzy C-Means (FCM) algorithm, referred to as SAGA-FCM, with the extended Genetic Grouping algorithm for clustering.

### Preparation before clustering: preprocessing, imputation, and dimensionality reduction

ScRNA-seq generates high-dimensional data, since each cell represents a multidimensional feature vector, and each dimension corresponds to a gene [6]. The curse of high dimensionality presents significant challenges for data analysis, including difficulties in modeling, increased computational time, susceptibility to noise, overfitting, and limited generalization [49]. To address these challenges, clustering analysis typically involves several preparatory steps. These include preprocessing, imputation, and dimensionality reduction.

Preprocessing methods such as gene filtering, log transformation, and normalization can help reduce noise and improve data quality. Tools such as Seurat (implemented in R) and Scanpy (Python) provide a variety of standardized preprocessing methods and have evolved into comprehensive pipelines of their own. These steps can enhance data quality and make subsequent clustering analyses more reliable.

Imputation is used to fill in missing data or address dropout zeros in scRNA-seq data and can impact clustering results. Several methods have been developed that successfully integrate imputation and clustering. In our survey, these include algorithms such as scDSC, DeepScena, AttentionAE-sc, scLEGA, and scDECL. However, there are also many specialized imputation algorithms such as GE-Impute [50]. This method is based on a graph-embedding neural network model that reconstructs cell–cell similarity networks for imputation. To provide a comprehensive survey, here we combined the GE-Impute imputation method alongside the classic Seurat clustering method. We also note that the developers of GE-Impute use Seurat clustering to assess the performance of their imputation method.

Dimensionality reduction is another step that is essential for achieving useful clustering outcomes. The primary goal of dimensionality reduction is to map high-dimensional data onto lower dimensional spaces while retaining critical variation and structural information within the dataset. All clustering algorithms in our survey began with dimensionality reduction before clustering was performed. Common techniques include principal component analysis (PCA), uniform manifold approximation and projection (UMAP), and various autoencoder algorithms.

## Datasets

### Dataset selection

To systematically evaluate the generalizability of biclustering and clustering algorithms, this study established a cross-species, cross-tissue benchmarking framework. To do so, 10 publicly available scRNA-seq datasets, including two model organisms (i.e. human and mouse) and encompassing critical biological processes including embryonic development (GSE45719), neural differentiation (GSE60361), and immune response (GSE81861) were selected. These datasets exhibit gradient scales (i.e. cell counts: 90–14 437; gene features: 19 161–57 241) and incorporate data variants processed using a variety of normalization methods, thereby simulating the technical heterogeneity encountered in real-world research. The reference cluster labels and cellular annotations, defined by the original authors in terms of their cellular origin, state, or developmental stages, were validated using multimodal approaches. Additional details regarding dataset specifications are provided in Table 1.

**Table 1:** Datasets

Datasets	Name	#cell	#gene	#clusters	Resource
Yan’s [51]	GSE36522	90	20 214	7	Human pre-implantation embryos and embryonic stem cells
Goolam’s [52]	E-MTAB-3321	124	41 480	5	Four-cell mouse embryos
Deng’s [53]	GSE45719	313	22 958	13	Mouse preimplantation embryos
Trapnell’s [19]	GSE52529	372	47 192	4	Human Skeletal Muscle Myoblasts (HSMM)
Usoskin’s [54]	GSE59739	864	25 333	9	The mouse lumbar dorsal root ganglion
Kolodziejczyk’s [55]	E-MTAB-2600	704	35 653	3	Three mouse ES cell
Li’s [56]	GSE81861	561	57 241	7	Primary colorectal tumors and matched normal mucosa
Zeisel’s [57]	GSE60361	3005	19 972	9	Two regions of the mouse cerebral cortex
Xin’s [58]	GSE81608	1600	39 851	8	Nondiabetic and type 2 diabetes organ donors
Chen’s [59]	GSE87544	14 437	28 234	47	Hypothalamus of mouse

### Methods of quantifying dataset properties

Since the complexity, diversity, and noise present in scRNA-seq datasets can affect the performance of biclustering or clustering [60], we investigated the performance of various biclustering and clustering methods in computing different dataset properties. These properties include sparsity, uncertainty, dispersion, complexity, difference, balance, and size. All concrete quantification methods for the dataset properties are described in Chapter 1 of the Supplementary Information. Moreover, the results of these six dataset assessments are shown in Table 2.

**Table 2:** Quantified results of the six dataset properties

Datasets	Sparsity	Uncertainty	Dispersion	Differences	Balance	Size
GSE36522	45.55%(low)	9.21(high)	25.03(med)	0.17(low)	0.11(low)	90 $\times $ 20 214(small)
E-MTAB-3321	68.56%(low)	5.31(med)	15.78(med)	1.14(med)	0.20(low)	124 $\times $ 41 480(small)
GSE45719	60.94%(low)	9.33(high)	40.11(med)	2.29(med)	0.06(high)	313 $\times $ 22 958(small)
GSE52529	85.79%(high)	3.49(med)	130.88(high)	0.01(low)	0.02(high)	372 $\times $ 47 192(med)
GSE59739	86.93%(high)	2.54(low)	137.09(high)	0.02(low)	0.09(med)	864 $\times $ 25 333(med)
E-MTAB-2600	70.83%(med)	6.09(med)	23.85(med)	6.17(med)	0.10(med)	704 $\times $ 35 653(med)
GSE81861	79.18%(med)	5.12(med)	56.99(med)	188.52(high)	0.09(med)	561 $\times $ 57 241(med)
GSE60361	81.21%(med)	1.21(low)	1.18(low)	0.38(low)	0.11(low)	3005 $\times $ 19 972(big)
GSE81608	85.63%(high)	3.29(med)	19.14(med)	0.01(low)	0.13(low)	1600 $\times $ 39 851(big)
GSE87544	93.36%(high)	1.25(low)	0.95(low)	126.91(high)	0.05(high)	14 437 $\times $ 28 234(large)

All metrics except “balance” show a positive correlation with the numerical values.

## Evaluation metrics

Two broad classes of metrics are used to evaluate the performance of clustering scRNA-seq data: internal and external metrics. Internal metrics evaluate clustering performance by measuring the separation and compactness of clusters, while external metrics measure clustering performance by estimating the relationship between the number of correctly clustered samples and the total number of samples. In this study, we selected three external metrics and two internal metrics to compare the performance of the biclustering and clustering methods. The three external metrics included Macro-F1 Score (F1), Adjusted Rand Index (ARI), and Normalized Mutual Information (NMI), while the two internal metrics included the Davies–Bouldin Index (DBI) and the Calinski–Harabasz (CH) metric. More information on each of these metrics can be found in Chapter 2 of the Supplementary Information.

## Comparative performance of biclustering and clustering methods for clustering scRNA-seq data

We conducted an extensive data analysis to evaluate the performance of five biclustering and 21 clustering methods studied here. First, we analyzed the performance of the biclustering and clustering methods across various dataset types one by one. This was performed to obtain quantifiable characterizations of dataset properties. Second, we summarized the performance of all biclustering and clustering methods using five overall evaluation metrics. Third, we conducted report the results of a category distribution analysis.

**Figure 1:**
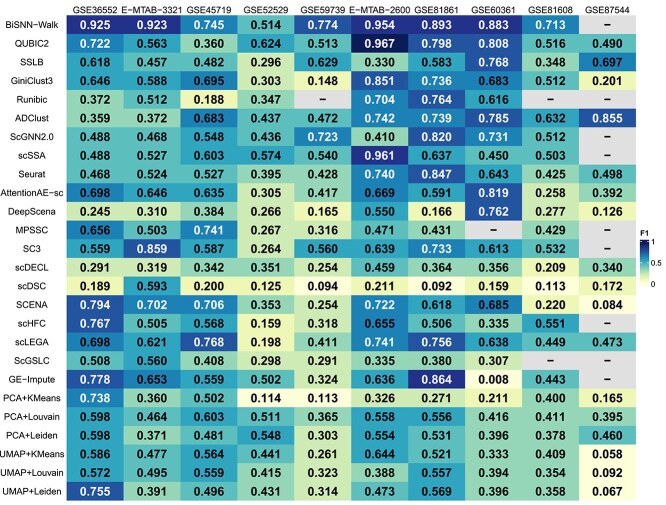
The result of the $F1$ score, where “-” indicates that the method is not applicable to this dataset.

### The performance of biclustering and clustering methods considering quantification results of dataset properties

We examined the clustering performance of five biclustering methods and 21 clustering methods using 10 datasets. Building upon the quantified dataset properties described in Chapter 3.2 and Table 2, we classified all 10 datasets six times each based on six different dataset properties. Thus we were able to analyze the results for each property individually. To visually compare clustering quality, we prioritized the $F1$ score as the primary evaluation criterion, and all results are illustrated in Fig. 1.

#### Sparsity

In datasets with low, medium, and high sparsity, we observed average $F1$ biclustering scores of 0.586, 0.756, and 0.477, respectively, while the average $F1$ scores of the clustering methods were 0.540, 0.530, and 0.351, respectively. These results indicate that the biclustering methods had superior performance across datasets with varying levels of sparsity. It is worth noting that the definitions of low, medium, and high sparsity used here do not imply ordinary data sparsity but are rather specifically defined for the inherently high dilution levels found in scRNA-seq data. Here, lower data sparsity indicates a higher number of expressed genes that were detected.

Moreover, differences in sparsity have important implications for both biclustering and clustering methods. For example, previous studies have shown that often only a small subset of genes are involved in cell regulation [61]. Clustering methods calculate cell similarity and clustering accuracy based on all genes, but this can make them susceptible to the influences of irrelevant genes. In contrast, biclustering methods are designed to identify and filter out key genes associated with specific cell subtypes. This minimizes the impact of irrelevant genes and leads to more accurate clustering results.

#### Uncertainty

For datasets with low, medium, and high uncertainty, the methods that achieved the highest $F1$ scores were QUBIC2 and BiSNN-Walk, both of which are biclustering methods. The average $F1$ scores for the biclustering methods in these categories exceeded those of the clustering methods by 0.020, 0.142, and 0.222, respectively. Therefore, we conclude that biclustering methods also outperform clustering methods across datasets with low, medium, and high uncertainty. This is because clustering methods can struggle to identify clear clustering boundaries in datasets with relatively uniform cell distributions, leading to lower accuracy. In contrast, biclustering methods focus on key genes, allowing them to modify the overall cell distributions used and to enhance the definition of clustering boundaries.

#### Dispersion

In low, medium, and high-dispersion datasets, biclustering scores consistently outperformed clustering method scores, with differences of 0.461 (0.348 for clustering), 0.637 (0.523 for clustering), and 0.643 (0.4 for clustering), respectively. The higher the dispersion in a dataset, the greater the variance between the expression level of an individual cell and the average for all cells, increasing the likelihood that cells with extreme expression levels are erroneously classified as outliers or as members of other clusters. Most clustering methods rely on intercell similarity or distance to determine cluster centers, which may cause decreased stability of clustering methods in high-dispersion datasets. Cells with extreme expression typically refer to those with either high expression, low expression, or unexpressed genes. In contrast, biclustering methods offer greater flexibility, but this can be a double-edged sword. It is generally useful that they selectively exclude extreme expression cells based on the current clustering stage, but the accuracy of such selections is not guaranteed.

#### Difference

We consider that biclustering methods are more suitable than clustering methods for datasets that are characterized by both low and high levels of difference. This is supported by not only the relatively high $F1$ scores achieved (i.e. 0.925 for a low-difference dataset and 0.893 for a high-difference dataset) but also the higher average $F1$ clustering scores generated.

In the medium difference scenario, biclustering methods yielded $F1$ values ranging from 0.188 to 0.967, with a mean of 0.621. In contrast, clustering methods exhibited $F1$ values ranging from 0.2 to 0.961, yielding a mean of 0.541. We further observed that biclustering algorithms also showed significantly better performance with a medium level of difference. This effect arises from disparities in probability distributions between clusters. A greater difference implies more distinct clustering boundaries. Therefore, biclustering methods are particularly effective for datasets with medium levels of difference. Clustering methods, on the other hand, modify the probability distributions, and may thereby reduce the inherent distinctiveness of these differences.

In addition, the difference between clusters is only one factor. Thus, when both inter- and intra-cluster differences are high or low simultaneously, determining precise cluster boundaries can be challenging. Researchers should therefore fully acknowledge all limitations imposed by the complexity of biological datasets.

#### Balance

In low-balance datasets, biclustering methods achieved $F1$ values ranging from 0.348 to 0.925, with a mean of 0.641. In contrast, clustering methods yielded $F1$ values ranging from 0.008 to 0.859, with a mean of 0.523. Overall, lower balance scores indicate a higher likelihood of rare cells being present in the dataset. This discrepancy may stem from certain clustering algorithms’ having challenges in identifying rare data. Given the higher average $F1$ values recorded here, we conclude that biclustering methods consistently perform better in identifying rare cell clusters.

For medium-balance datasets, biclustering methods generated average $F1$ values of 0.67, whereas the clustering methods average 0.554. Thus, we conclude that biclustering methods are better for handling medium-balance datasets.

In high-balance datasets, biclustering methods average an $F1$ value of 0.461, compared with clustering methods at 0.336. Notably, the ADClust method achieves the highest score of 0.855 in the GSE87544 dataset, surpassing the best biclustering score of 0.774 by 0.081. Therefore, we assert that clustering methods demonstrate greater efficacy in managing balanced datasets overall. This superior performance is attributed to their ability to effectively capture the inherent structures and patterns present in such datasets.

#### Size

On datasets of small, medium, big, and large sizes, the average $F1$ scores of biclustering methods were 0.046, 0.164, 0.217, and 0.165 (respectively) higher than those ofclustering methods. Therefore, we further conclude that the overall performance of the biclustering methods compared is superior to that of clustering methods. However, in large-sized datasets, the highest $F1$ score (0.855) was achieved by ScGNN 2.0, exceeding the highest biclustering algorithm by 0.158. We assert that ScGNN 2.0 is the most suitable algorithm for handling large datasets. In addition, it is noteworthy that when dealing with datasets greater than $10^{9}$, half of both the biclustering and clustering methods were unable to be run.

Overall, the average $F1$ scores of biclustering algorithms are slightly higher than those of clustering algorithms across all scenarios. However, the average $F1$ score reflects a specific aspect of performance and may not provide a comprehensive evaluation of overall performance. In the following section, we conduct a more thorough comparison using additional evaluation metrics.

### Evaluating biclustering and clustering methods based on aggregate performance on five metrics

**Figure 2:**
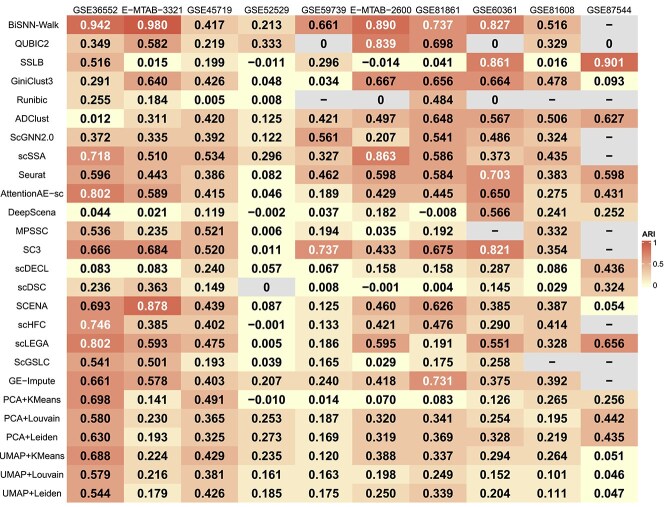
The result of the $ARI$ score, where “-” indicates that the method is not applicable to this dataset, and the occurrence of “0” is due to the fact that the method generates only one result for this dataset.

**Figure 3:**
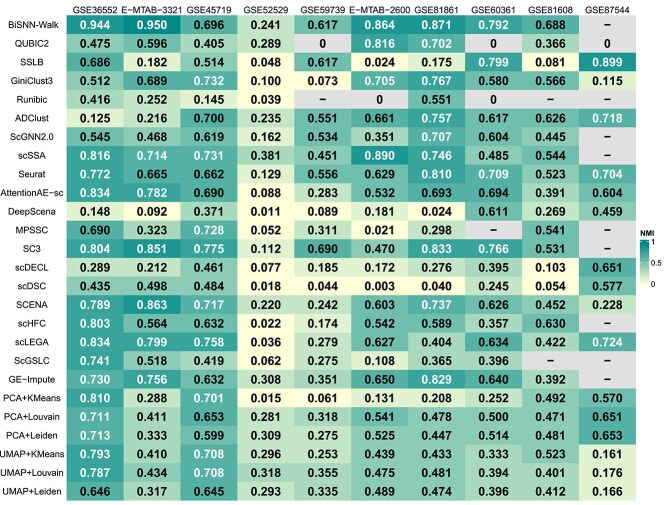
The result of the $NMI$ score, where “-” indicates that the method is not applicable to this dataset, and the occurrence of “0” is due to the fact that the method generates only one result for this dataset.

**Figure 4:**
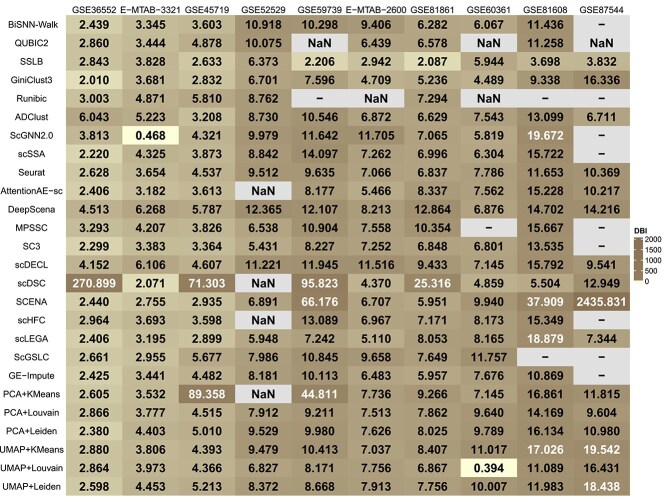
The result of the $DBI$ score, where “-” indicates that the method is not applicable to this dataset, and the occurrence of “NaN” is due to the fact that the method generates only one result for this dataset.

**Figure 5:**
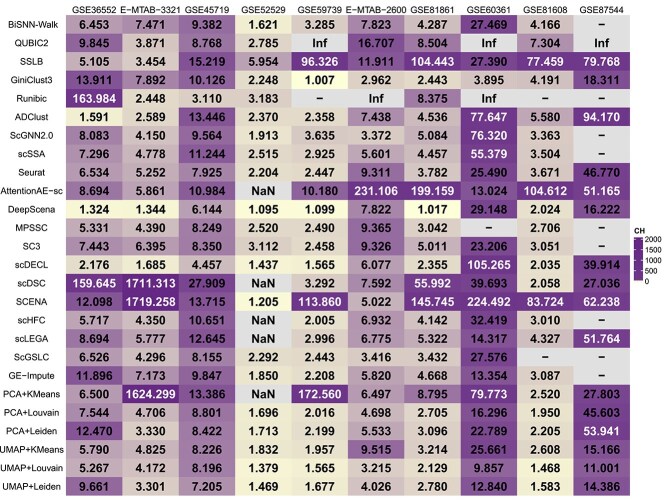
The result of the $CH$ score, where “-” indicates that the method is not applicable to this dataset, and the occurrence of “Inf” is due to the fact that the method generates only one result for this dataset.

We conducted an aggregate analysis of the clustering performance of five biclustering methods and 21 clustering methods on 10 datasets using three internal metrics and two external metrics. The performance results for each of the five metrics are shown in Figs 1, 2, 3, 4, and 5.

For three external metrics, the average scores of all biclustering methods perform better for all clustering methods’ on two external evaluation. Across all datasets involved in the survey, the average $F1$ score for all biclustering methods was 0.603, while for clustering methods’, it was 0.465. Biclustering methods attain an average $ARI$ value of 0.376, while clustering methods achieve 0.331. Additionally, the average score of all clustering methods performs better on the $NMI$ metric. The average $ARI$ value for biclustering is 0.447, whereas for clustering, it is 0.464.

For the two internal evaluation metrics, biclustering algorithms outperformed clustering algorithms in terms of DBI, while clustering algorithms performed better in terms of CH. Specifically, the average $DBI$ value for all biclustering methods was 5.814, while for all clustering methods it was 23.286. Here, a smaller $DBI$ value indicates better clustering. In contrast, the mean $CH$ value for all biclustering methods was 19.631, while the mean for all clustering methods was 43.721. A larger $CH$ indicates better clustering.

Overall, across the five evaluation metrics, biclustering methods achieved higher average scores on three metrics, while clustering methods outperform on the remaining two. However, the superiority of biclustering methods over clustering methods cannot be conclusively determined solely based on these results. Our survey serves as a reference framework that emphasizes the necessity of performing holistic evaluations when selecting optimal methods for distinct data types. For example, when mining datasets with unknown annotations, we recommend clustering methods because of their robustness for exploratory analysis. Conversely, when conducting deep investigations of partially annotated datasets, biclustering methods are more likely to uncover latent data features.

### Category distribution analysis of biclustering and clustering methods

**Figure 6:**
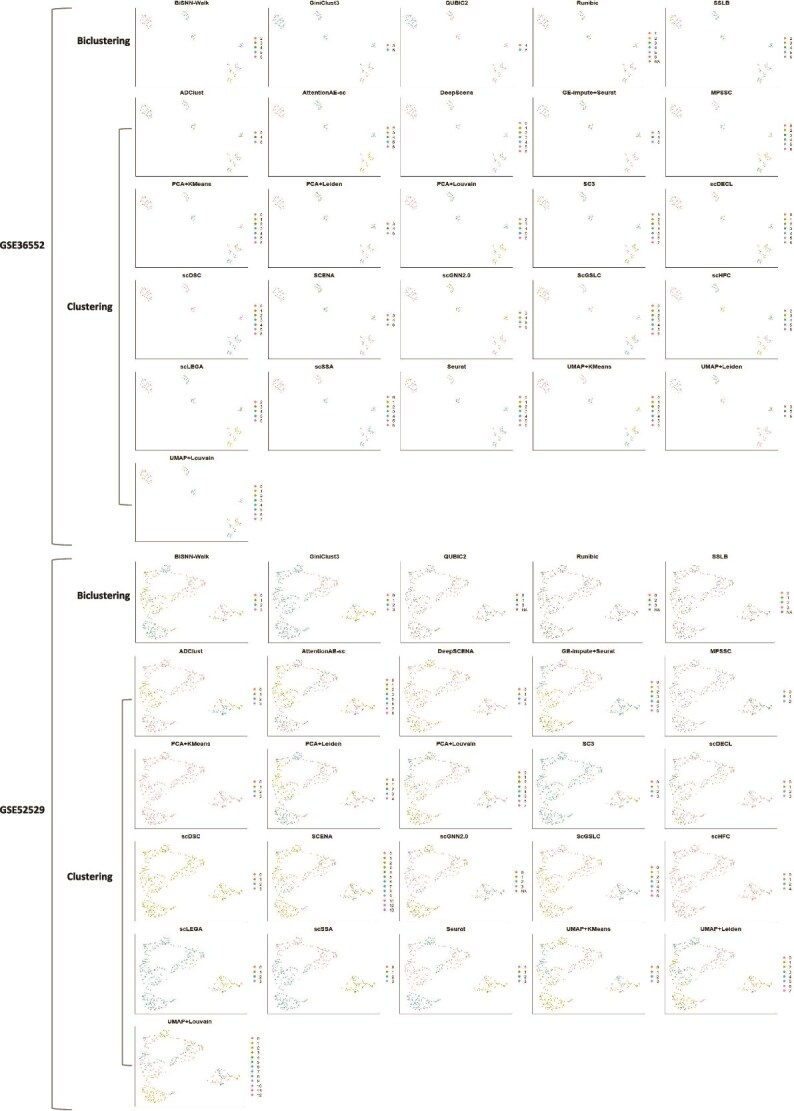
Visualization plots including each cell cluster, where the gray parts represent that the corresponding cells are lost during the clustering process, and the blanks represent that the corresponding methods cannot be applied to the dataset yet.

To visually present the category distribution of each cell cluster, we visualized the clustering results for each cell cluster. Figure 6 shows the visualization results for two datasets as an example. The visualization results for all datasets can be found in Chapter 3 of the Supplementary Information. Here, we analyzed category distribution from three perspectives. We also collated the specifics of these five biclustering and 21 clustering methods in Table 3.

**Table 3:** Comparison of different methods

Methods	$AvgF1$	Have NA?	Need K?	Speed	Language	Time complexity	Year
BiSNN-Walk	0.814	yes	no	fast	R	$O(n^{2}m)$	2017
QUBIC2	0.636	yes	no	slow	C++	$O(nm^{2})$	2019
SSLB	0.521	yes	no	slow	R	$O(nm+ms)$	2021
GiniClust3	0.536	yes	no	fast	Python	$O(n^{2}m)$	2020
runibic	0.500	yes	no	very slow	R	$O(n^{2}m)$	2018
ADClust	0.608	no	no	fast	Python	$O(n^{2}m)$	2020
scGNN2.0	0.571	yes	yes	moderate	Python	$O(inm)$	2020
scSSA	0.587	no	yes	moderate	Python	$O(nm^{2})$	2020
Seurat	0.550	no	no	fast	R	$O(n^{2}m)$	2021
AttentionAE-sc	0.543	no	no	fast	Python	$ O(inm)$	2023
DeepScena	0.325	no	no	fast	Python	$ O(inm)$	2023
MPSSC	0.477	no	no	fast	MATLAB	$O(n^{3})$	2018
SC3	0.594	yes	no	fast	R	$O(nm^{2})$	2017
scDECL	0.329	no	yes	moderate	Python	$ O(inm)$	2023
scDSC	0.195	no	yes	fast	Python	$O(n^{2}m)$	2023
SCENA	0.514	no	no	fast	R	$O(n^{2})$	2021
scHFC	0.485	no	no	fast	R+Python+MATLAB	$O(nm^{2})$	2021
scLEGA	0.575	no	no	fast	Python	$O(m^{3})$	2024
ScGSLC	0.380	no	no	moderate	Python	$O(n^{3})$	2021
GE-Impute+Seurat	0.530	no	no	slow	Python+R	$O(n^{2}m)$	2022
PCA+KMeans	0.320	no	yes	fast	Python	$O(nm^{2})$	-
PCA+Louvain	0.488	no	yes	fast	Python	$O(nm^{2})$	-
PCA+Leiden	0.462	no	yes	fast	Python	$O(nm^{2})$	-
UMAP+KMeans	0.429	no	yes	fast	Python	$O(n^{2})$	-
UMAP+Louvain	0.415	no	yes	fast	Python	$O(n^{2})$	-
UMAP+Leiden	0.425	no	yes	fast	Python	$O(n^{2})$	-

In terms of cluster boundaries, we found that biclustering methods typically produced clearer cluster boundaries than clustering methods. From Fig. 6 in two-dimensional space, we can directly observe that in most cases, the cells within each cluster obtained by biclustering methods remain tightly connected, with a certain distance between clusters and with clear cluster boundaries. It is important to note that this two-dimensional visualization is only as a reference, since distances and relationships may change when mapping from a higher dimensional space to a two-dimensional space.

In terms of cell integrity, clustering methods generally preserve cell integrity well, whereas some biclustering methods may cause cell loss. As shown in the third column of Table 3, clustering methods rarely exhibit missing values, while the five biclustering methods used in our experiments contain missing values (NA). Additionally, the visualization plots of QUBIC2 and SSLB in Fig. 6 both show high percentages of NA values. This occurs because many biclustering methods select only highly expressed genes during the initial gene clustering step. Since these genes are only expressed in specific cell subsets, some cells may be excluded prior to cell clustering.

In terms of cell categories, the major challenge faced by biclustering and clustering methods is determining the number of cell categories ($k$-value). Biclustering and clustering methods apply different strategies to determine the optimal $k$-value. For biclustering methods, the $k$-value is determined by mining local coexpression patterns. For example, statistical significance tests can be used to select coexpressed gene pairs, sparse constraints can be applied to control the number of factors, overlapping clusters can be merged, or automatic division can be based on local density thresholds. In contrast, clustering methods typically determine the $k$-value by relying on the integrity of the global data structure, as characterized by internal evaluation metrics (e.g. the CH index or silhouette coefficient), modular optimization in graph clustering, statistical tests (e.g. eigenvalue difference analysis), or biological priors (e.g. marker gene expression patterns).

### Time complexity of biclustering and clustering methods

We summarized the time complexity of biclustering and clustering methods, as shown in the seventh column of Table 3. Here, $n$ represents the number of cells, $m$ represents the number of genes, $i$ represents the number of iterations, and $s$ represents the number of genes after gene filtering. According to Table 3, the time complexity of both the biclustering algorithms and clustering algorithms is nonlinear. Although biclustering methods still require clustering of both genes and cells, the time complexity of biclustering methods is still not generally higher than that of all clustering algorithms.

## Discussion

Based on the analysis in the previous chapter, both biclustering and clustering methods exhibit distinct specificities. This chapter summarizes the strengths and weaknesses of biclustering and clustering methods, respectively, and proposes some improvement strategies.

### Advantages, drawbacks, and improvement suggestions for biclustering methods

The advantage of biclustering methods lies in their ability to capture local consistency. Given that a specific gene or experimental condition can involve multiple biological pathways, leading to the potential assignment of a gene or condition to multiple clusters simultaneously [61], capturing local consistency is crucial. Biclustering methods typically tend to produce more accurate clustering results and clearer cluster boundaries, likely due to their consideration of both cell and gene characteristics simultaneously.

Biclustering methods have four main disadvantages. First, the degree of refinement in biclustering is often unclear. As noted in Section 5.3, biclustering methods usually yield more detailed results compared with clustering methods, typically subdividing cells into more groups. Although this finer granularity may reveal intricate biological processes, it can also complicate biological annotation and potentially overlook common features across larger groups. The granularity of clustering is closely related to the number of method iterations. Too many iterations can lead to overfitting and excessive fragmentation, whereas insufficient iterations may not produce the desired clustering outcomes. Implementing adaptive iteration methods, which adjust the number of iterations based on convergence metrics or clustering stability assessments, is a potential solution, although this approach requires further investigation.

Second, most biclustering methods may lead to cell loss, as mentioned in the previous Section 5.3. Future research must address this issue to prevent such loss. Researchers should consider revising biclustering strategies to avoid selecting few highly expressed genes and their associated cells, and could implement strategies to identify cell loss after each biclustering round.

Third, biclustering methods should be made more user-friendly. Some methods require users to input parameters such as the number of clusters ($K$), which can be a challenging task for datasets lacking annotations. Existing studies have shown that the selection of input parameters can affect clustering accuracy [62, 63]. Therefore, we recommend that method developers provide automated or data-driven approaches to assist in setting these parameters, thereby markedly reducing the user’s burden. Employing cross-validation based on data characteristics or heuristic methods to determine these thresholds will ensure that the selected clustering models are both robust and interpretable.

Finally, the computational time required for biclustering methods needs improvement. In our experiments, the three slowest methods were all biclustering methods, as evidenced in the seventh column of Table 3. Therefore, balancing accuracy and computational complexity will be a key consideration in the future development of biclustering methods.

### Advantages, disadvantages, and improvement suggestions for clustering methods

Clustering methods are widely used and generally more stable. Most of these methods preserve cell integrity, making them particularly suitable for scenarios where complete cell information is required. Additionally, clustering methods typically offer faster computational speeds, reducing time costs.

However, future research must address two key issues in addition to improving clustering accuracy. Notably, considering column 6 in Table 3, half of the tested clustering methods require the prior introduction of true labels. This is due to the methods being based on semi-supervised learning and needing to learn existing classification rules before performing further clustering. Many data types lack fixed standards or rules to determine a cluster as a specific cell type. Cell annotation often relies on experience, which may introduce biases and implicit understandings. This limitation affects their applicability and prevents their use in unannotated cell populations. Therefore, it is essential to carefully consider the applicability conditions and the significance of the developed methods in future clustering studies.

The second issue is the generalizability of clustering methods. Typically, our results show that certain methods excel when applied to specific datasets but perform poorly when applied to others. The inherent heterogeneity and diversity in scRNA-seq data lead to substantial structural differences between datasets. Consequently, developing a clustering method that excels universally across all scRNA-seq datasets is challenging. Future research should focus on balancing characteristics between different species and sample datasets to improve clustering performance across methods. Additionally, conducting detailed analyses of datasets and identifying customized methods that best suit the characteristics of each dataset may become a primary research direction. Establishing a unified approach to integrate the best clustering methods also represents a valuable strategy.

## Future outlook

Considering current trends in scRNA-seq development, we explore two critical directions to guide future research: adapting to the era of big data, and integrating multi-omics and spatiotemporal omics data for subsequent research.

### Adapting to the era of big data

The volume of scRNA-seq data is expected to continue expanding. Advances in sequencing technology now allow for greater sequencing depth and the ability to handle more samples, potentially capturing dynamic changes in cells across various time points and spatial locations. Such high-resolution spatiotemporal data are crucial for constructing aggregated biological, spatiotemporal maps and will guide future research directions. As our understanding of the mechanisms underlying complex diseases deepens, future research may involve analyses of data from multiple samples, time points, and even organizational perspectives to fully elucidate disease progression and biological complexity. For example, in cancer research, systematic tracking of sequencing data from early, intermediate, and late disease stages is highly valuable. Relying on a single sample alone does not provide a complete picture of the disease; thus, aggregate sequencing analyses of tumor microenvironments, primary foci, and metastatic sites are necessary to understand tumor heterogeneity and dissemination mechanisms. Consequently, the volume of sequencing data will likely increase exponentially. To manage this influx, there is an urgent need to develop downstream analysis methods capable of handling larger and more complex datasets effectively. Additionally, the computational resources and time required for big data analysis will increase substantially. Future research must balance accuracy with computational efficiency to ensure that large datasets are processed accurately within a reasonable timeframe, placing higher demands on data analysis.

### Integrating multi-omics and spatiotemporal omics data for subsequent research

The sparsity and technical noise inherent in scRNA-seq data often lead to biases in cell subtype identification, prompting researchers to adopt multi-omics integration strategies to improve cell-type resolution. For instance, Cobolt employs a variational autoencoder to jointly model scRNA-seq and ATAC-seq data [64], significantly enhancing the identification of rare cell subpopulations; Seurat v4’s WNN algorithm constructs multimodal similarity maps to refine clustering resolution [32]. However, most existing methods rely on feature concatenation strategies, failing to adequately address differences in detection sensitivity and dynamic ranges across omics platforms or resolve intrinsic conflicts in cross-omics data integration. The deep learning architectures and causal inference method may provide new prospects to address these challenges. The deep learning architectures are capable of simultaneously modeling spatial proximity, temporal continuity, and multi-omics associations, thereby constructing interpretable regulatory network models. The causal inference method could identify core regulatory factors driving cell-state transitions (e.g. transcription factor enhancer-metabolite trios) from regulatory network models. As Stuart *et al*. [65] emphasized, the ultimate goal of clustering extends beyond cell classification to unraveling the regulatory logic underlying phenotypic hetheterogeneity—a task requiring closed-loop integration of computational models and experimental validation. While precise cell-type identification marks the first step, future advancements are poised to transform single-cell research from static atlases into four-dimensional analyses of living systems, decoding spatiotemporal dynamics across developmental and disease trajectories.

## Conclusion

The emergence of scRNA-seq technology has facilitated major advancements in biology. The present survey provides a comprehensive comparison and analysis of five prominent biclustering methods and 21 clustering methods developed in recent years. Through this comparative analysis, we identified the most suitable biclustering or clustering methods for various dataset properties, summarized the strengths and weaknesses of these methods and potential improvement strategies, and offered insights for future research in clustering analysis.

For partially annotated datasets or when exploring local consistency, it is advisable to prioritize biclustering methods. When it is necessary to preserve complete cell information, improve computational speed, or handle unknown datasets, clustering methods are recommended.

Despite the development of numerous effective biclustering and clustering methods, major challenges remain. Researchers must improve the scalability of these methods to address the challenges of large-scale data. Integrating multi-omics data to perform clustering analysis from multiple perspectives, as well as improving the practical utility of clustering through biological interpretation and validation, is crucial to advance the field.

**Key Points**

- Biclustering for local consistency and partially annotated datasets: for datasets that are partially annotated or when exploring local consistency, it is advisable to prioritize biclustering methods, since these methods can effectively capture intricate patterns within subsets of data.
- Clustering for preserving complete information and scalability: clustering methods are suitable when the goal is to preserve complete cell information, enhance computational efficiency, or handle unknown datasets. These methods are generally more scalable and efficient for large datasets.
- Challenges and future directions: despite the development of numerous effective biclustering and clustering methods, major challenges remain. Researchers must enhance the scalability of both methods to address the increasing scale of data challenges. Integrating multi-omics data and improving the biological interpretation and validation of clustering results are also crucial for advancing this field.

## Supplementary Material

Supplementary-Revise2_elaf010
