# Supplementary material for: A survey of biclustering and clustering methods in clustering different types of single-cell RNA sequencing data
Source: Brief Funct Genomics. 2025 Aug 10;24:elaf010. doi: 10.1093/bfgp/elaf010 (PMC12342763; doi:10.1093/bfgp/elaf010)
Supplement: Supplementary-Revise2_elaf010 [file supplementary-revise2_elaf010.docx]

**Supplementary**

1. **Quantification methods for the dataset properties**

1.1 Quantification method for dataset sparsity

In scRNA-seq dataset, the majority of gene in scRNA-seq dataset remain unexpressed in most cells. Consequently, there exist quantities of zero values meaning unexpressed in scRNA-seq data, which poses challenges for clustering analysis.

This sparsity hinders the accurate capture of similarities between samples, and renders clustering outcomes highly sensitive to minor fluctuations in the dataset, which in turn reduces the stability of the clustering results.

Evaluating the sparsity of the dataset is thus crucial for an aggregate assessment. To quantify this sparsity, we have opted to measure the percentage of zero elements in the dataset to represent the sparsity.

Based on the calculation results, we define sparsity below 70% as low, sparsity between 70%-85% as medium, and sparsity above 85% as high.

1.2 Quantification method for dataset uncertainty

Information entropy stands as a fundamental concept within the realm of information theory that is used to measure the distribution and uncertainty of data, and its mathematical expression formula is:

$$H\left( X \right)=-\sum_{i=1}^{n} p\left( x_{i} \right)\cdot\log_{2}\left( p\left( x_{i} \right) \right)$$

Where $p\left( x_{i} \right)$ represents the probability distribution of each possible event $x$ in random variable $X$.

It can be seen that information entropy focuses on characterizing the distribution of non-zero elements within a dataset. As the value of information entropy increases, so does the uncertainty of the dataset in question. In contrast, the lower the value of the information entropy, the lower the uncertainty of the dataset. This phenomenon arises from the central role of probability distributions within the information entropy. A higher information entropy signifies a more uniformly distributed dataset, wherein the probabilities of all elements appearing are more evenly balanced. Under such circumstances, it becomes challenging to ascertain the likelihood of any specific outcome, akin to the act of flipping a coin. When the probabilities of getting heads or tails are equal, predicting the outcome of the coin toss accurately becomes notably uncertain, thereby exemplifying a classic scenario of high uncertainty.

Based on the calculation results, we define uncertainty as low if it is below three, medium if it is between three and seven, and high if it is above seven.

1.3 Quantification method for dataset dispersion

Dispersion can help us assess the distribution of a dataset. A low dispersion indicates that the data points tend to be concentrated in a certain area, while a high dispersion indicates a wider distribution of data points. There are a number of statistical methods to measure dispersion, we have chosen to use the standard deviation as the measure, whose mathematical calculation is as follows:

$$s=\sqrt{\frac{1}{K}\sum_{i=1}^{K} \left( x_{i}-x \right)^{2}}$$

Calculating the standard deviation of the data helps us to detect outliers or outliers present in the dataset, which may also have an impact on clustering.

According to the calculation results, we define the value of dispersion below 10 as low, the value of dispersion between 10 and 100 as medium, and the value of dispersion above 100 as high.

1.4 Quantification method for differences among cell clusters

Kullback-Leibler divergence[1] is a statistic used to measure the difference between two probability distributions, so we used it to measure the difference among different clusters. The higher the value of KL scatter, the greater the difference between clusters of cells, and the greater the difference between cells, the easier it is to cluster. The calculation method is as follows:

$$KL=\sum_{i=1}^{K} \sum_{j\neq i}^{K} \left( \left( P_{i}\left( x \right)+\epsilon\right) \right.\log_{2}\left( \frac{P_{i}\left( x \right)+\epsilon}{P_{j}\left( x \right)+\epsilon} \right)$$

Where$K$represents the total number of clusters,$P_{i}\left( x \right)$and$P_{j}\left( x \right)$denote the probability densities of cell clusters $i$ and $j$ respectively, calculated using the kernel density estimation method. $\epsilon$ is a small smoothing constant used to prevent zero values, and we take $e^{-8}$ as the smoothing constant.

According to the calculation results, we define the differences values below 1 as low, differences between 1-100 as medium, and differences above 100 as high.

1.5 Quantification method for balance among cell clusters

In our study, we select a series of single-cell RNA sequencing datasets with known truth labels. These datasets exhibit diversity in the distribution of true labels, with some datasets having relatively uniform label distributions, while others contain rare cell clusters. The identification of rare cell clusters has consistently been a significant challenge in the analysis of scRNA-seq data, as these clusters often encompass subtypes or cellular states of particular importance to the research. To comprehensively assess the performance of different clustering methods in dealing with this challenge, we employ the following methods to quantify the distribution of the true labels:

$$SD=\sqrt{\frac{1}{K}\sum_{i=1}^{K} \left( P_{i}-P \right)^{2}}, P=\sqrt{\frac{1}{N}\sum_{i=1}^{N} P_{i}}$$

Where $P_{i}$ represents the proportion of cells in the i-th cluster relative to the total cells. The larger the value, the more unbalanced in cell number proportions among different clusters.

Based on the calculation results, we define the value of balance greater than 0.1 as low, balance between 0.07 and 0.1 (including 0.1) as medium, and balance below 0.07 as high.

1.6 Quantification method for dataset size

We measure the size of a dataset by the product of the number of cells and the number of genes. We consider the order of magnitude$10^{7}$as "small size", $1\times10^{8}$ to $5\times10^{8}$ as "medium size", $5\times10^{8}$ to $1\times10^{9}$ as "big size", and the order of magnitude $10^{9}$ as "large size".

1. **Evaluation Metrics**

We chose five metrics to evaluate the performance of these nine clustering methods, including three external metrics (Macro-F1 Score, Adjusted Rand Index, Normalized Mutual Information) and two internal metrics (Davies-Bouldin lndex, Calinski-Harabaz Index) . External metrics are used to evaluate the clustering performance by comparing the clusters used as a benchmark with the clusters of the method. Internal measures evaluate clustering performance without a benchmark by examining the separation and compactness of the clusters.

2.1 External Metrics

(1) F1

We chose the Macro-F1 Score (hereinafter referred to as $F1$) as the evaluation metric, which is the harmonic mean of precision and recall. This metric does not take into account variations in cluster sizes, focusing on the average performance across all clusters. The value of $F1$ is between 0 and 1, the closer to 1 the better the performance, and the formula is as follows:

$$F1=\frac{1}{K}\sum_{i-1}^{K} \left( \frac{2}{\frac{1}{Precision}+\frac{1}{Recall}} \right)$$

(2) ARI

Rand Index ($RI$) is a common metric for assessing congruence, but it has a limitation in terms of its penalty for dissimilarity. Adjusted Rand Index ($ARI$) is developed to address this limitation, providing a more precise quantification of the congruence between true labels and cluster assignments [2]. $ARI$ values range between -1 and 1, with higher values indicating a stronger congruence.

$$ARI=\frac{RI-E\left( RI \right)}{\max\left( RI \right)-E\left( RI \right)}$$

(3) NMI

Normalized Mutual Information ($NMI$) is also a metric for assessing the congruence between true labels and cluster assignments. It measures the mutual dependence between two random variables from an information-theoretic perspective. The range of $NMI$ values is from 0 to 1, with values closer to 1 indicating better clustering performance.

$$NMI=\frac{2\times I\left( T;C \right)}{H\left( T \right)+H\left( C \right)}$$

Where $T$ respresents true labels, and $C$ respresents cluster assignments. $H\left( T \right)$ respresents the entropy of the set $T: H\left( T \right)= -\begin{matrix} \sum_{i=1}^{\left| T \right|} P\left( i \right)\log_{2}P\left( i \right) \end{matrix}$. $I\left( T;C \right)$ represents mutual information: $\begin{matrix} I\left( T;C \right)=H\left( T \right)-H\left( T|C \right) \end{matrix}$.

2.2 Internal Metrics

(1) DBI

Davies-Bouldin lndex ($DBI$) is a relatively stable and robust internal metric that takes into account both the similarity between clusters and the differences within clusters. A smaller $DBI$ value indicates greater similarity within clusters and larger differences between clusters, signifying better clustering performance. In detail, the formula for $\mathrm{DBI}$ is:

$$DBI=\frac{1}{K}\sum_{i=1}^{K} \max_{j\neq i}\left( \frac{s_{i}+s_{j}}{d\left( c_{i},c_{j} \right)} \right)$$

Where $s_{i}$ and $s_{j}$ is the average distance between data points within cluster i and cluster j. $d\left( c_{i},c_{j} \right)$denotes the Euclidean distance between $c_{i}$ (the centroid of cluster $i$) and $c_{j}$ (the centroid of cluster $j$).

(2) CH

Calinski-Harabaz Index ($CH$) evaluates the quality of clustering by comparing the relationship between within-cluster variance and between-cluster variance. Low variance indicates small differences, while high variance indicates significant differences. The mathematical description is as follows:

$$CH=\frac{\sum_{k=1}^{K} n_{k}\left\| c_{k}-c \right\|^{2}}{\sum_{k=1}^{K} \sum_{i=1}^{n_{k}} \left\| d_{i}-c_{k} \right\|^{2}}\times\frac{n-K}{K-1}$$

The numerator on the left side represents the intra-cluster distance, while the denominator represents the inter-cluster distance. In this formula, $n_{k}$ represents the number of cells within the k-th cluster. $c_{k}$ represents the mean of all data points within the k-th cluster, which is also known as the centroid of that cluster. $d_{i}$ represents the i-th data point within the k-th cluster. $c$ represents the mean of all data points. A higher $CH$ value indicates a better clustering outcome.

1. **Visualization of Cell Clusters**

In this section, we present detailed visualizations of the category distribution for each cell cluster across all datasets.

For E-MTAB-3321 dataset：


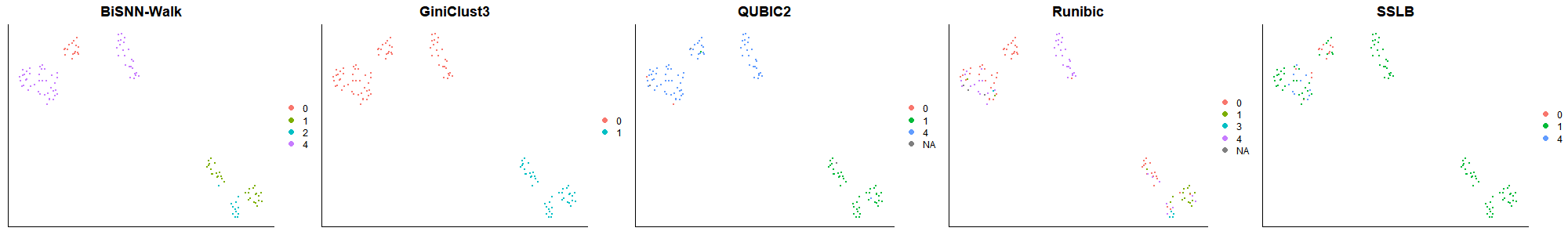


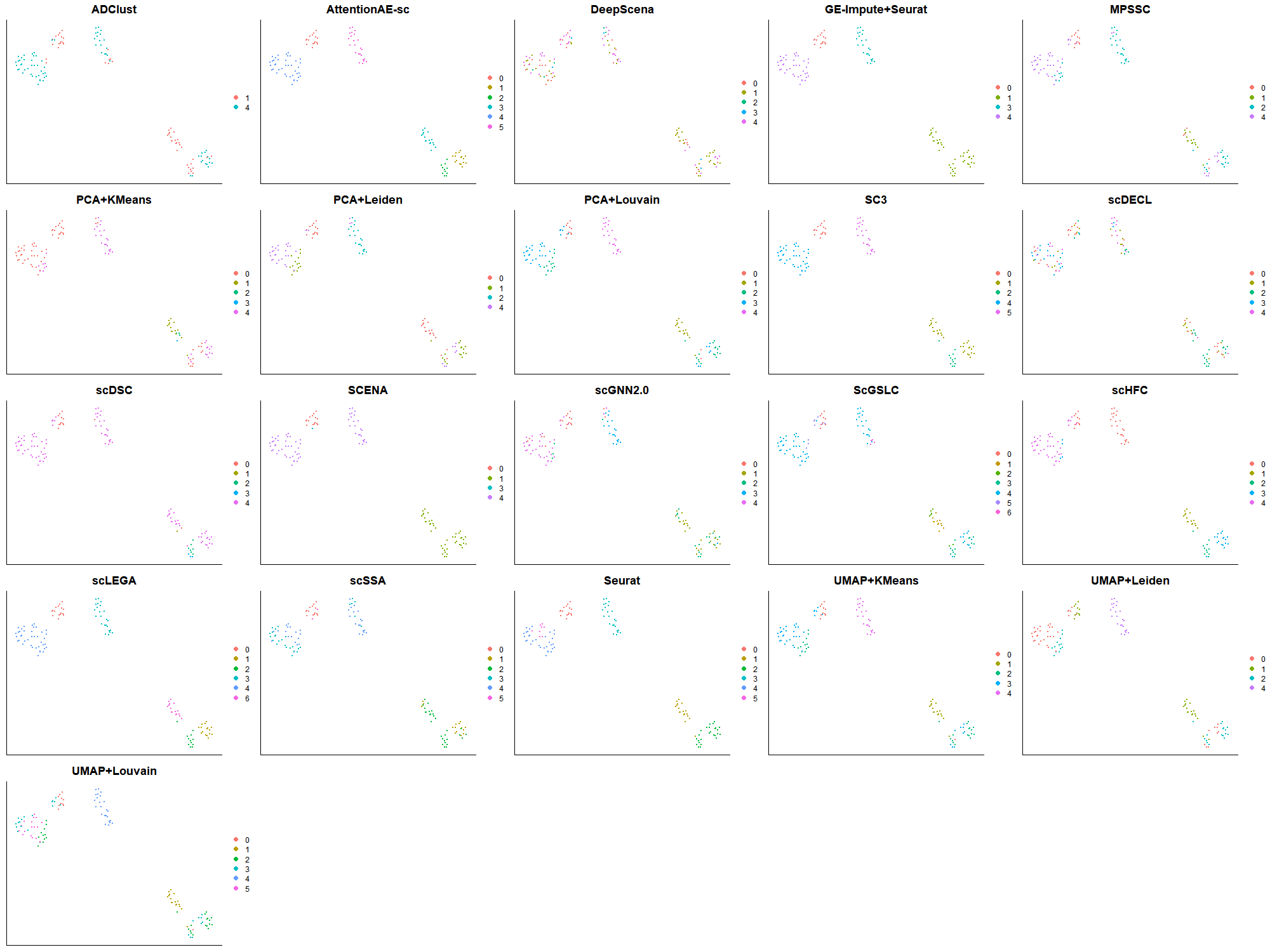


For GSE45719 dataset：


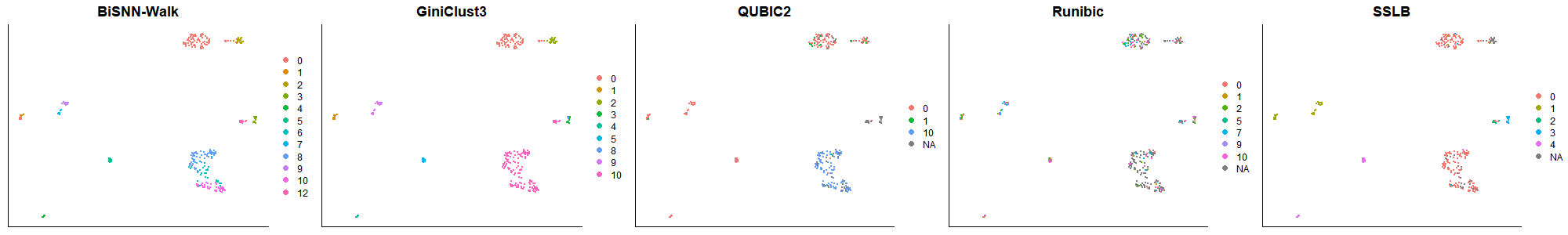

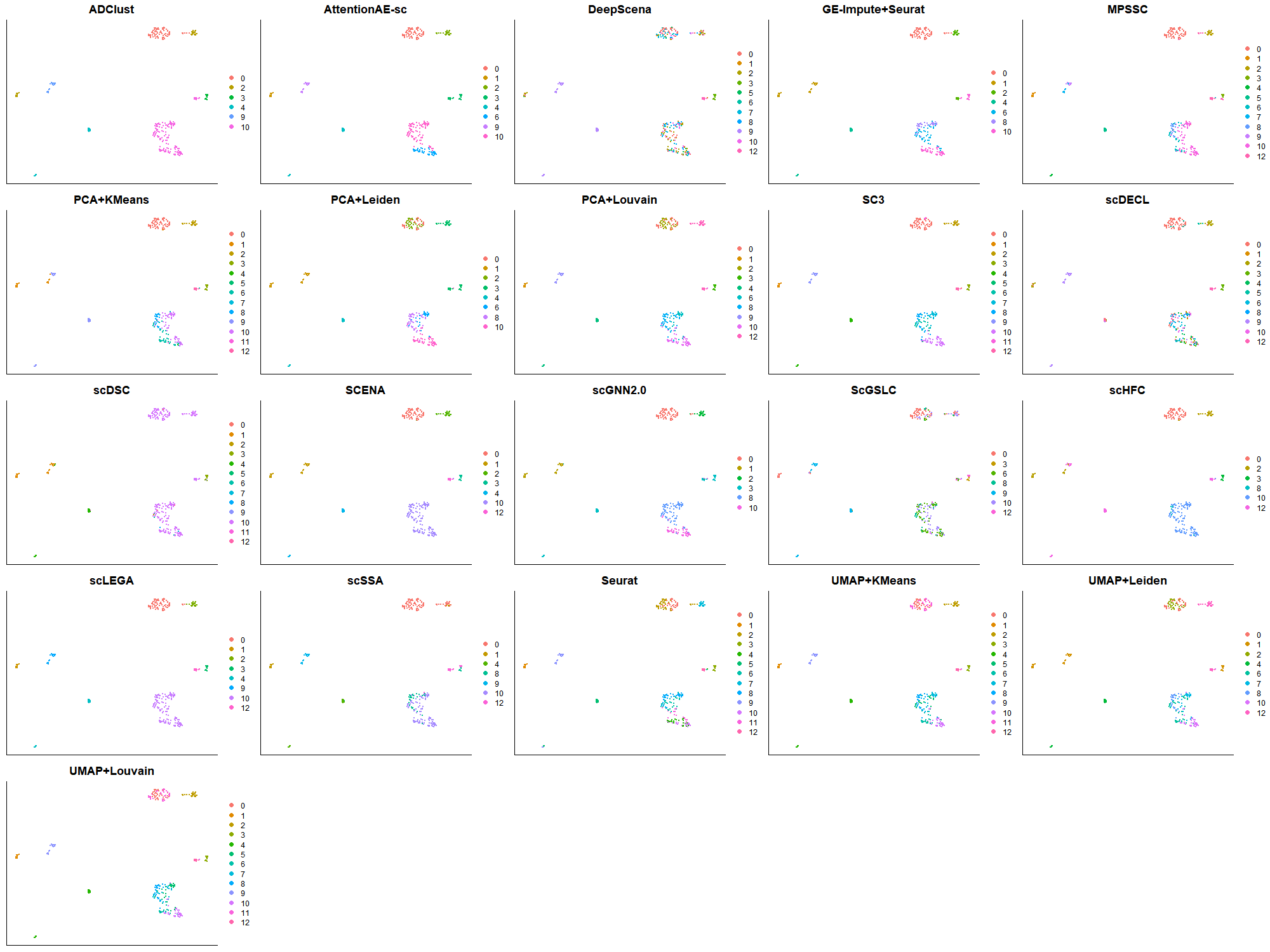


For GSE59739 dataset：


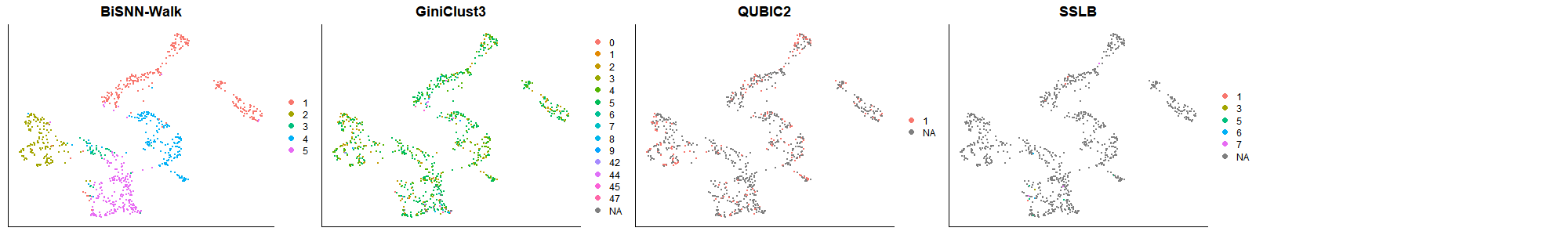


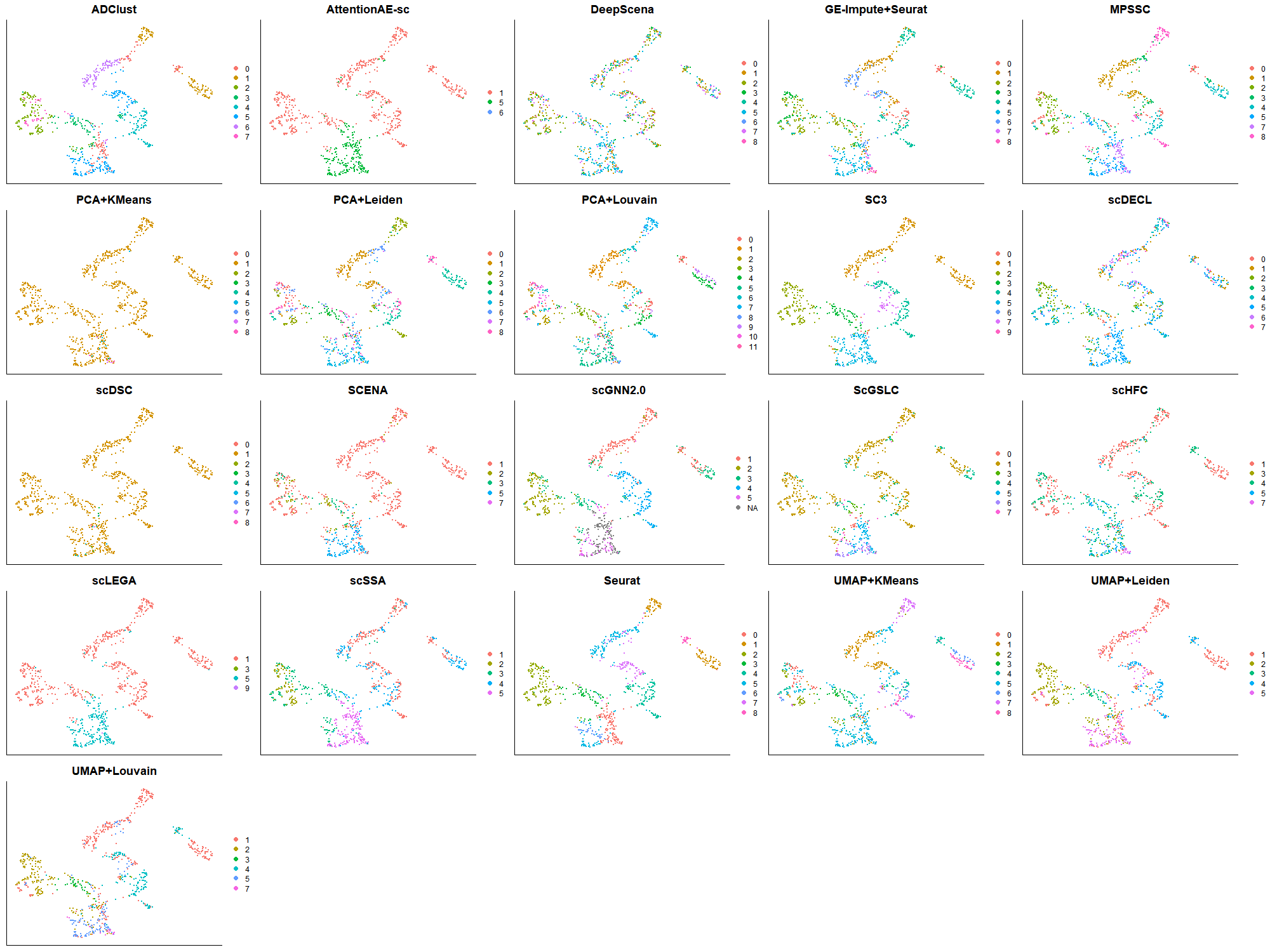


For E-MTAB-2600 dataset：


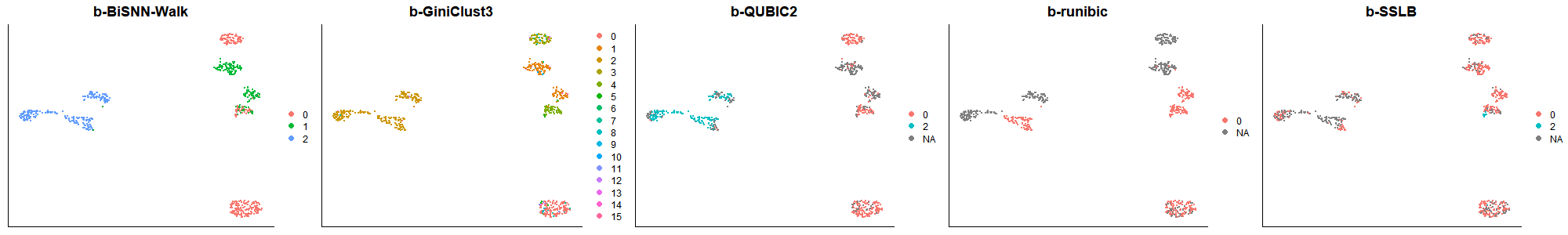


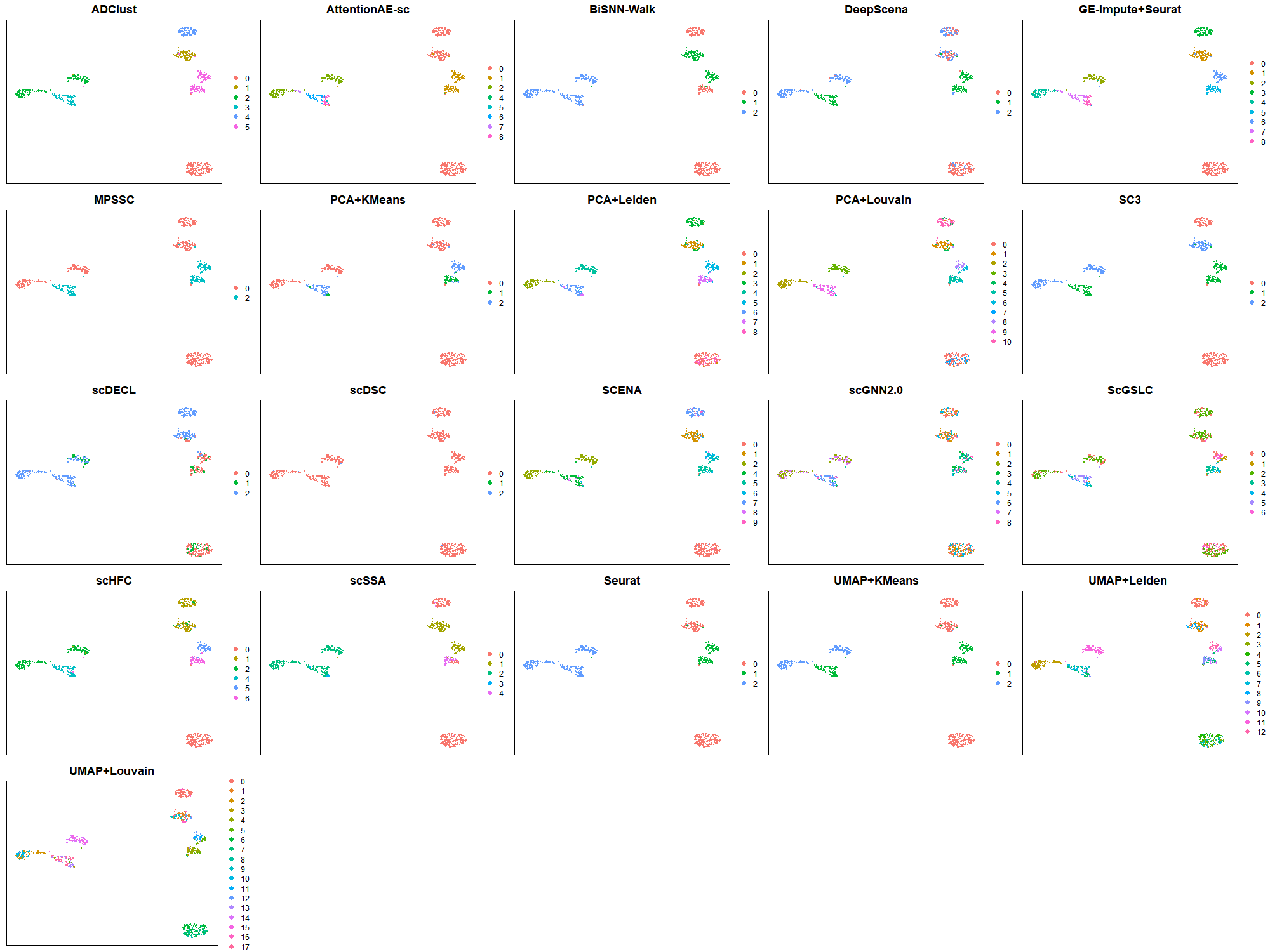


For GSE81861 dataset：


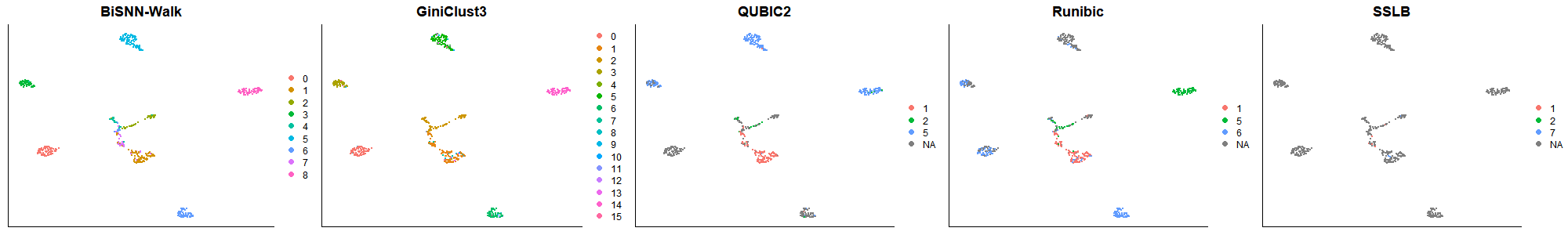


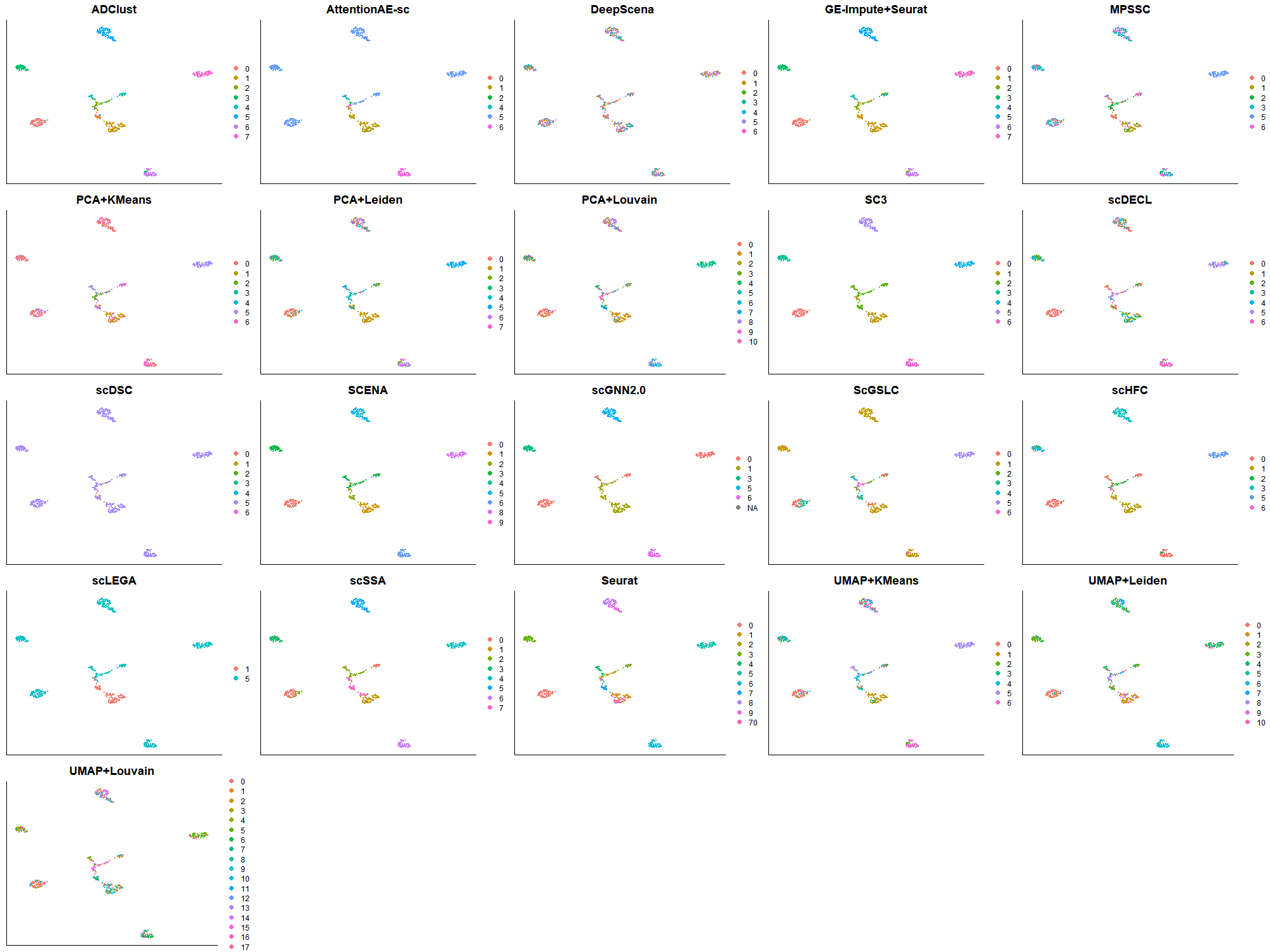


For GSE60361 dataset：


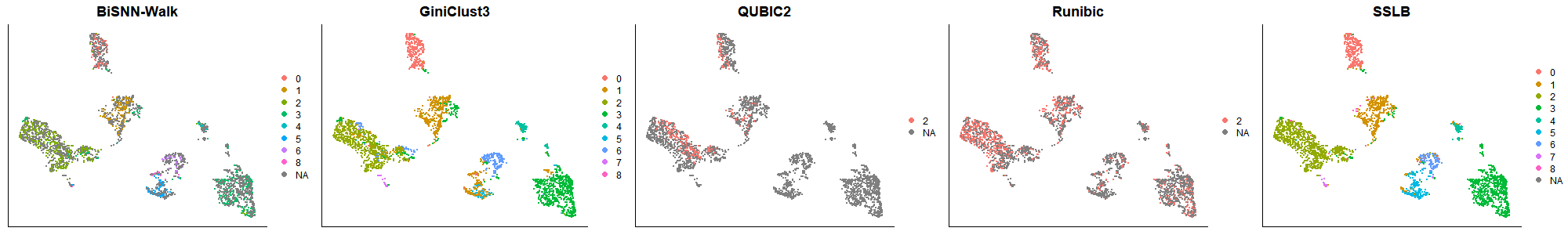


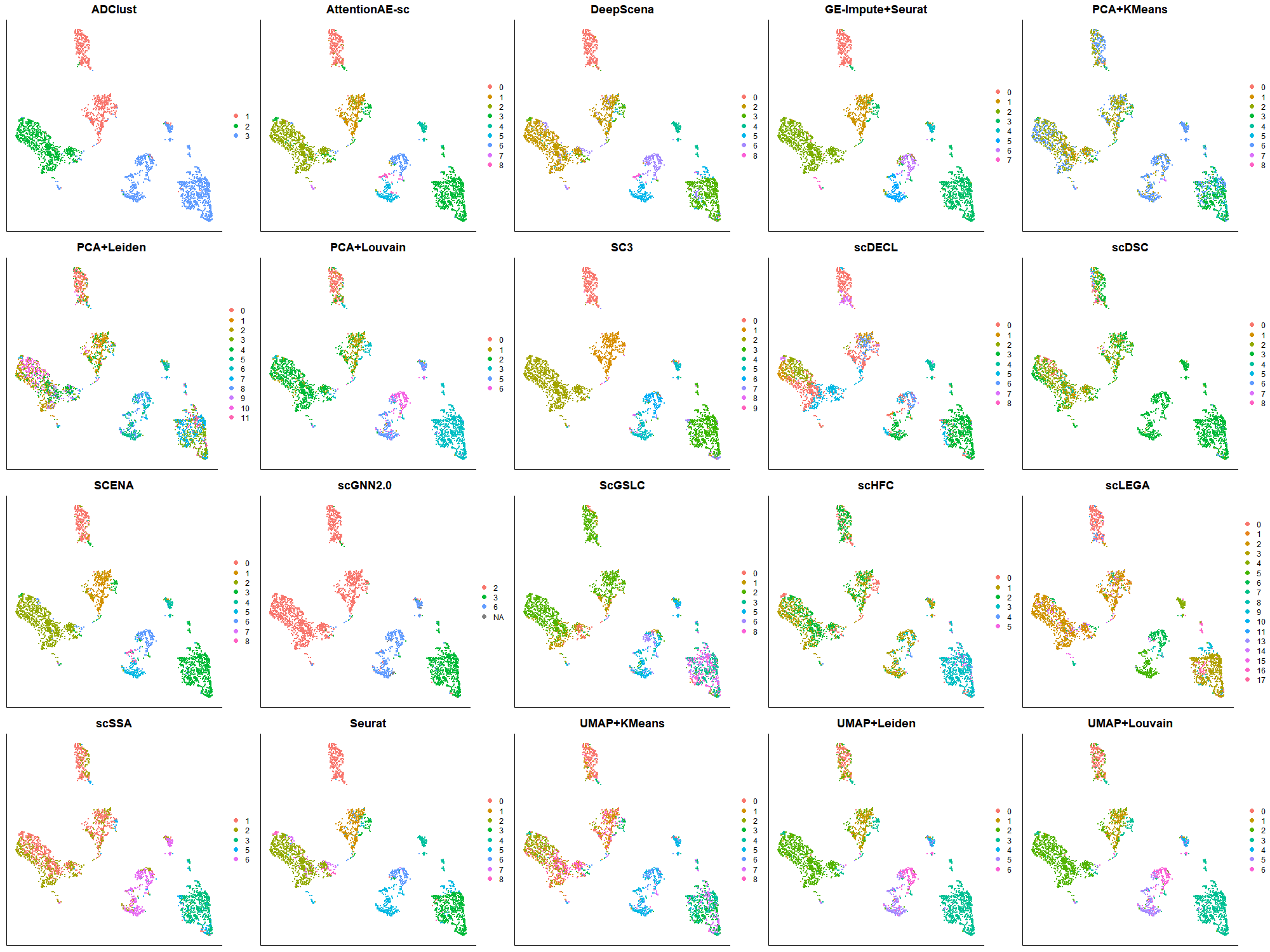


For GSE81608 dataset：


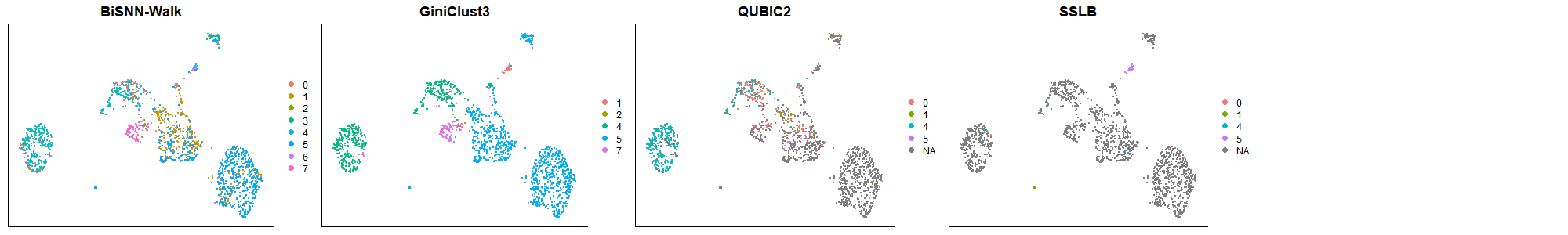


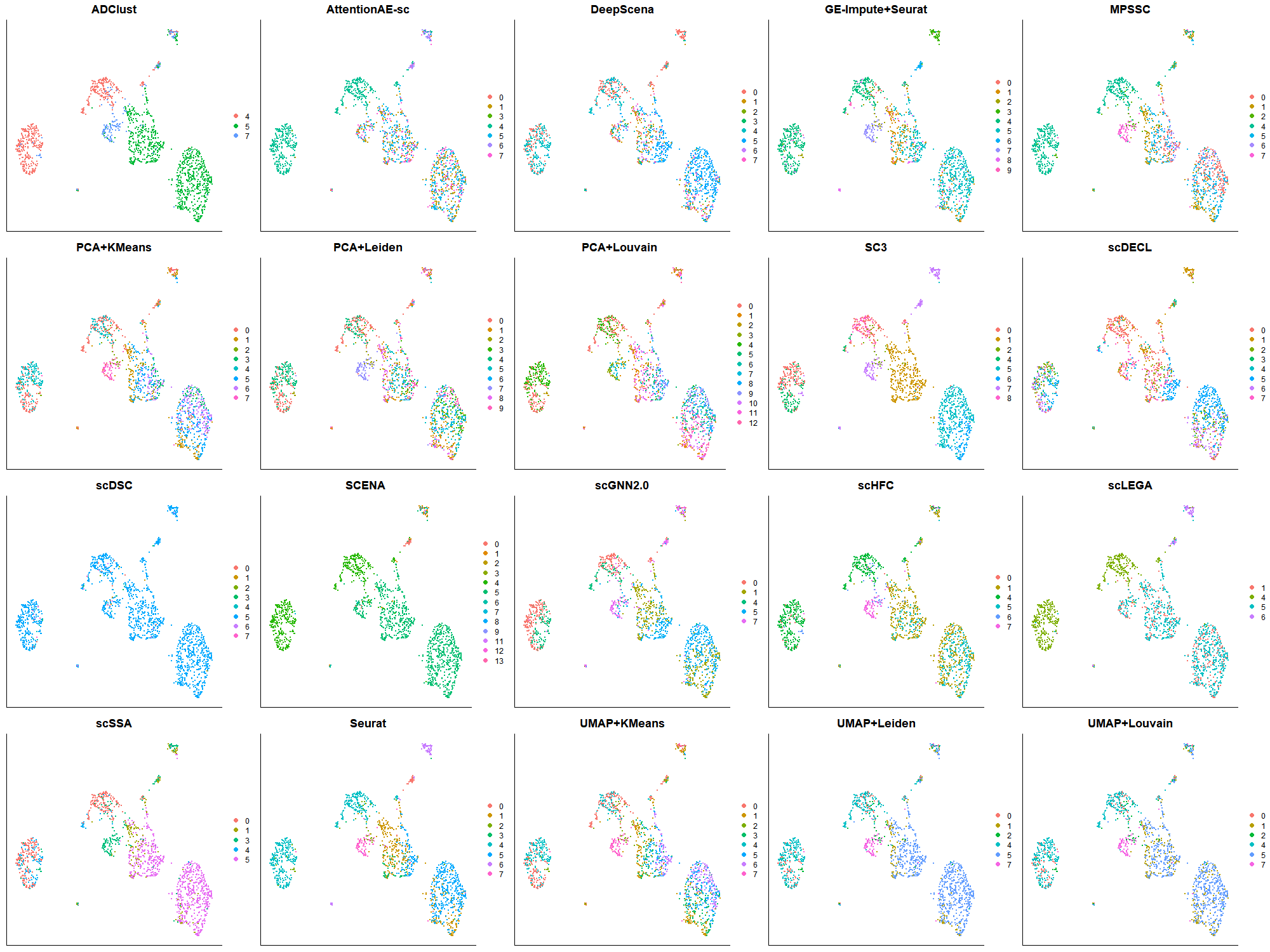


For GSE87544 dataset：


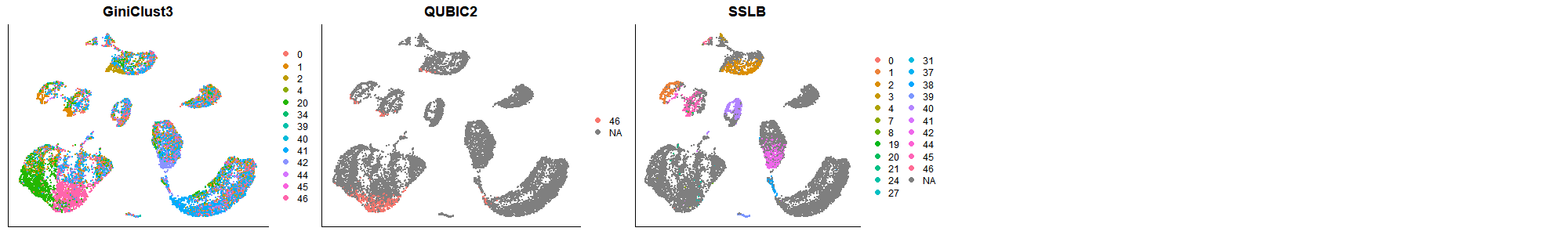


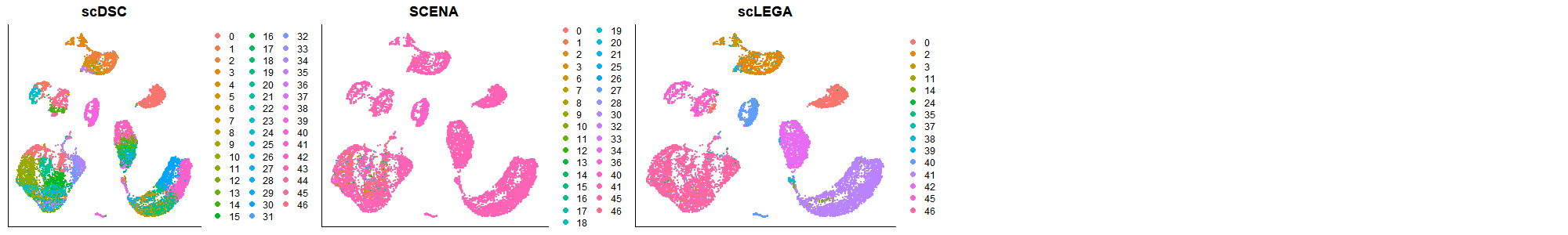

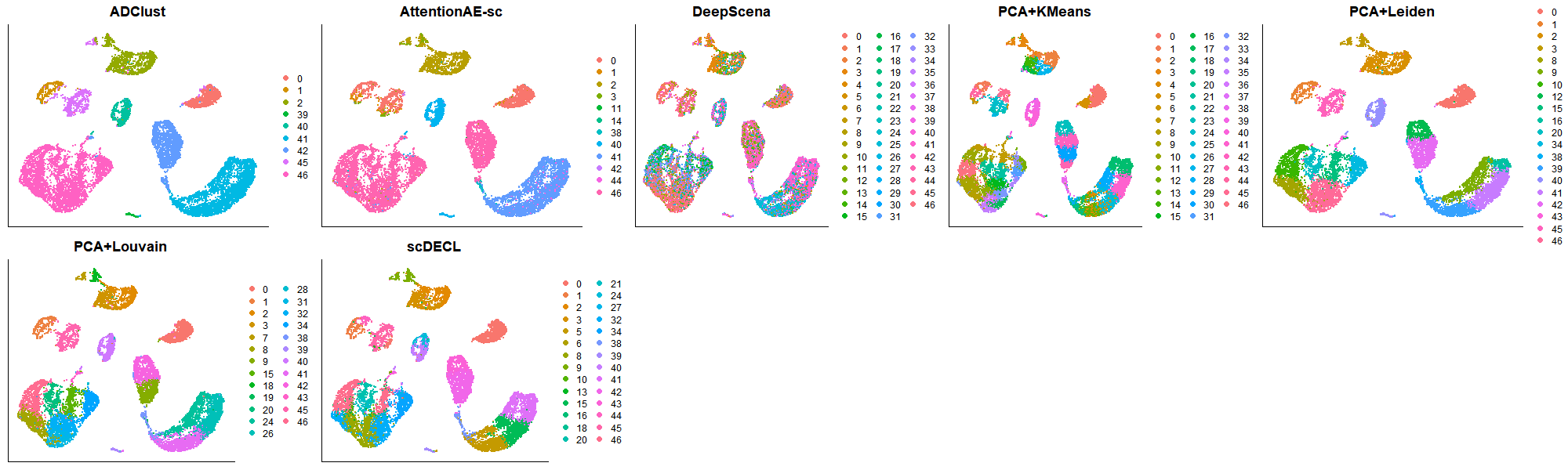


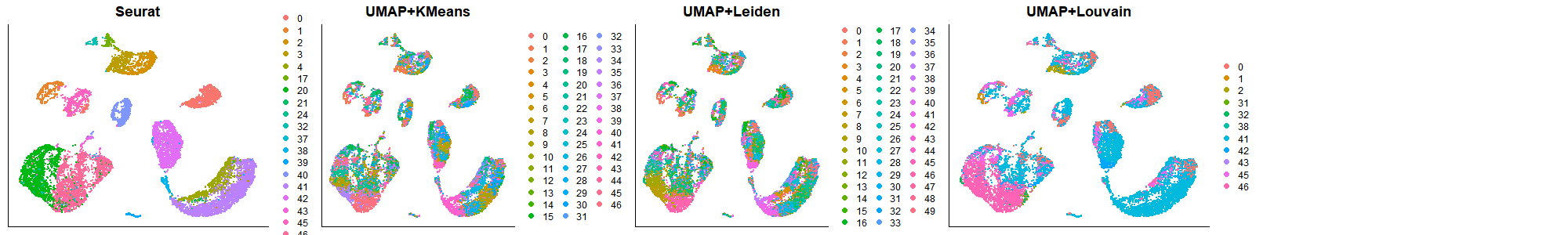


**References**

1. Kullback S, Leibler RA. On information and sufficiency. The annals of mathematical statistics. 1951 Mar 1;22(1):79-86.
2. Jorge M Santos and Mark Embrechts. On the use of the adjusted rand index as a metric for evaluating supervised classification. In International conference on artificial neural networks, pages 175–184. Springer, 2009.

Dear editor: We submit our manuscript entitled “A Survey of Biclustering and Clustering methods in Clustering Different Types of Single-Cell RNA

Sequencing Data” to Briefings in Functional Genomics for publication. Our research investigates the clustering and biclustering methods applied to single-cell RNA sequencing (scRNA-seq) data, which we believe will be of significant interest to the journal’s readers. The innovation points and significance of our study are primarily reflected in the following aspects: Our study identifies the most suitable methods for different types of datasets, assisting users in selecting the most appropriate methods based on specific data characteristics. We summarize the strengths and weaknesses of both biclustering and clustering methods, providing valuable suggestions for potential improvements in these methods. Additionally, our research offers insights and recommendations for future clustering research. All authors have seen themanuscript and approved to submit to your journal. Thank you very much for your attention and consideration. Sincerely yours, Xiaoqi Tang.
